# Supplementary material for: New Emerging Targets in Cancer Immunotherapy: The Role of B7-H3
Source: Vaccines (Basel). 2024 Jan 5;12(1):54. doi: 10.3390/vaccines12010054 (PMC10820813; doi:10.3390/vaccines12010054)
Supplement: Supplementary file 1 [file vaccines-12-00054-s001.zip › vaccines-2743887-SI.pdf]

**Supplementary Table S1.** Expression patterns of B7-H3 in solid malignancies.

| **Malignancy** | **B7-H3 expression** | | | | **Tumor cell expression** | **Cellular localization** | | | **Stromal expression** | **Reference** |
|---|---|---|---|---|---|---|---|---|---|---|
| | Total | Weak | Moderate | Strong | | Nucleus | Cytoplasm | Cell membrane | | |
| Hepatocellular carcinoma | 93.8% | 23.8% | 37.1% | 32.9% | | N/A | N/A | N/A | 86.2 % | [71] |
| | 88.6% | 9.6% | 51.6% | 38.7% | N/A | N/A | N/A | N/A | N/A | [72] |
| | 90.5% | 58.7% | N/A | 41.3% | N/A | N/A | N/A | N/A | N/A | [73] |
| Pancreatic adenocarcinoma | 66% | 68% | 19.7% | 12.3% | N/A | N/A | N/A | N/A | N/A | [74] |
| | 63% | 73% | 27% | 0% | 21% | N/A | N/A | N/A | 63% | [28] |
| | 88% | N/A | N/A | N/A | N/A | N/A | N/A | N/A | N/A | [75] |
| | 93.2% | 29% | N/A | 71% | 93.2% | N/A | N/A | N/A | N/A | [76] |
| **Gastric adenocarcinoma** | 76% | 44% | moderate or strong in 56% | | 18% | N/A | N/A | N/A | 82% | [29] |
| | 58.8% | N/A | N/A | N/A | N/A | N/A | N/A | N/A | N/A | [77] |
| | 78% | N/A | N/A | N/A | N/A | N/A | N/A | N/A | N/A | [78] |
| | 85% | 20% | 65.7% | 14.3% | High expression in 18.7% | N/A | N/A | N/A | High expression in 62.7% | [79] |
| **Colorectal carcinoma** | 81.6% | 47.9% | 23.9% | 28.1% | N/A | N/A | N/A | N/A | N/A | [80] |
| | 87% | 12.6% | 19.8% | 67.6% | N/A | 27% | Cytoplasmic or membrane expression in 86% | | 77% | [26] |
| **Cervical cancer** | 94% | 62.8% | N/A | N/A | N/A | N/A | N/A | N/A | 94% | [30] |

| | | | | | | | | | | |
|---|---|---|---|---|---|---|---|---|---|---|
| | 72.2% | N/A | N/A | 36.7% | N/A | N/A | N/A | N/A | N/A | [81] |
| **Endometrial serous carcinoma** | 93% | N/A | N/A | N/A | N/A | N/A | N/A | N/A | N/A | [82] |
| | 77.7% | 77.7% | N/A | N/A | N/A | N/A | N/A | N/A | 60.6% | [83] |
| **Ovarian cancer** | 93% | 93% | N/A | N/A | N/A | N/A | N/A | N/A | Scattered | [84] |
| **SCLC** | 64.9% | N/A | N/A | N/A | N/A | Predominantly cytoplasmic and membranous | | | N/A | [85] |
| | 64.49% | N/A | N/A | N/A | N/A | N/A | N/A | N/A | N/A | [86] |
| **NSCLC** | 69.5% | N/A | N/A | N/A | N/A | N/A | N/A | N/A | N/A | [87] |
| | 80.4% | Predominant | N/A | N/A | N/A | Predominantly cytoplasmic and membranous | | | Scattered | [88] |
| | 90% | N/A | 35.45% | 30.91% | 23.64% | Predominantly cytoplasmic and membranous | | | N/A | [89] |
| **Breast cancer** | 56.8% | N/A | N/A | N/A | N/A | Predominantly cytoplasmic and membranous | | | N/A | [90] |
| | 90.6% | N/A | N/A | N/A | N/A | N/A | N/A | N/A | N/A | [91] |
| RCC | 95.1% | 17.4% | N/A | N/A | N/A | N/A | N/A | N/A | 95.1% | [92] |
| | 96.9% | 96.9% | N/A | N/A | N/A | 4% | 79.1% | 96.9% | N/A | [27] |
| Prostate cancer | 47% | N/A | 25.5% | 44.9% | 28.7% | Predominantly membranous | | | N/A | [93] |
| **Neuroblastoma** | 82% | 30.6% | 35.3% | 34.1% | N/A | N/A | N/A | N/A | N/A | [94] |
| | 94.4% | 17.6% | 52.9% | 29.4% | N/A | N/A | N/A | N/A | N/A | [95] |
| **Melanoma** | N/A | N/A | N/A | N/A | N/A | N/A | N/A | N/A | N/A | N/A |

Abbreviations: N/A: not available/not reported.
